# Population variability in thermal performance of pre-spawning adult Chinook salmon

**DOI:** 10.1093/conphys/coad022

**Published:** 2023-05-03

**Authors:** Jacey C Van Wert, Brian Hendriks, Andreas Ekström, David A Patterson, Steven J Cooke, Scott G Hinch, Erika J Eliason

**Affiliations:** Department of Ecology, Evolution & Marine Biology, University of California, Santa Barbara, Santa Barbara, CA 93106-9620, USA; Pacific Salmon Ecology and Conservation Laboratory, Department of Forest and Conservation Sciences, The University of British Columbia, Vancouver, BC V6T 1Z4, Canada; Fish Ecology and Conservation Physiology Laboratory, Department of Biology, Carleton University, Ottawa, ON K1S 5B6, Canada; Department of Biological and Environmental Sciences, University of Gothenburg, 41390 Gothenburg, Sweden; Fisheries and Oceans Canada, Science Branch, Cooperative Resource Management Institute, School of Resource and Environmental Management, Simon Fraser University, Burnaby, BC V5A 1S6, Canada; Fish Ecology and Conservation Physiology Laboratory, Department of Biology, Carleton University, Ottawa, ON K1S 5B6, Canada; Pacific Salmon Ecology and Conservation Laboratory, Department of Forest and Conservation Sciences, The University of British Columbia, Vancouver, BC V6T 1Z4, Canada; Department of Ecology, Evolution & Marine Biology, University of California, Santa Barbara, Santa Barbara, CA 93106-9620, USA

**Keywords:** thermal biology, temperature, Oncorhynchus, local adaptation, intraspecific variability, conservation, climate change, Chinook salmon, aerobic metabolic scope

## Abstract

Climate change is causing large declines in many Pacific salmon populations. In particular, warm rivers are associated with high levels of premature mortality in migrating adults. The Fraser River watershed in British Columbia, Canada, supports some of the largest Chinook salmon (*Oncorhynchus tshawytscha*) runs in the world. However, the Fraser River is warming at a rate that threatens these populations at critical freshwater life stages. A growing body of literature suggests salmonids are locally adapted to their thermal migratory experience, and thus, population-specific thermal performance information can aid in management decisions. We compared the thermal performance of pre-spawning adult Chinook salmon from two populations, a coastal fall-run from the Chilliwack River (125 km cooler migration) and an interior summer-run from the Shuswap River (565 km warmer migration). We acutely exposed fish to temperatures reflecting current (12°C, 18°C) and future projected temperatures (21°C, 24°C) in the Fraser River and assessed survival, aerobic capacity (resting and maximum metabolic rates, absolute aerobic scope (AAS), muscle and ventricle citrate synthase), anaerobic capacity (muscle and ventricle lactate dehydrogenase) and recovery capacity (post-exercise metabolism, blood physiology, tissue lactate). Chilliwack Chinook salmon performed worse at high temperatures, indicated by elevated mortality, reduced breadth in AAS, enhanced plasma lactate and potassium levels and elevated tissue lactate concentrations compared with Shuswap Chinook salmon. At water temperatures exceeding the upper pejus temperatures (T_pejus_, defined here as 80% of maximum AAS) of Chilliwack (18.7°C) and Shuswap (20.2°C) Chinook salmon populations, physiological performance will decline and affect migration and survival to spawn. Our results reveal population differences in pre-spawning Chinook salmon performance across scales of biological organization at ecologically relevant temperatures. Given the rapid warming of rivers, we show that it is critical to consider the intra-specific variation in thermal physiology to assist in the conservation and management of Pacific salmon.

## Teaser

Warming water temperatures threaten aquatic species and impact economies and cultures. Yet, vulnerability can vary within a species. We assessed the thermal performance of two populations of pre-spawning adult Chinook salmon and found that an interior summer-run population of Chinook salmon performed better at warmer temperatures than a coastal fall-run population. Current river temperatures already exceed the functional warming tolerances of both populations and highlight the value of physiological studies in supporting management decisions.

## Introduction

Pacific salmon (*Oncorhynchus* spp*.*) are fundamental to the ecosystems, economy and culture of the Northeast Pacific (Naiman *et al.,* 2002; Jacob *et al.,* 2010; Gislasin *et al.,* 2017). Their lifetime fitness depends on their ability to migrate from the Pacific Ocean to natal freshwater spawning grounds on finite energy reserves to then spawn and die (i.e. they are semelparous; Mesa and Magie, 2006; Farrell *et al.,* 2008). Salmon return to their natal spawning grounds with high fidelity, which in turn maintains many genetically and geographically distinct populations. These populations experience different environmental conditions during their upriver migration depending on when they enter the river and where they spawn, resulting in populations that are locally adapted to their migration conditions (Lee *et al.,* 2003b; Eliason *et al.,* 2011). However, warming rivers are causing mass mortalities of adult spawning salmonids across species and populations throughout their ranges (Gilhousen, 1990; Schreck *et al.,* 1994; Keefer *et al.,* 2008; Scholz *et al.,* 2011; Martins *et al.,* 2012; Bowerman *et al.,* 2018; von Biela *et al.,* 2022). Therefore, there is an immediate need to better understand the thermal physiology of Pacific salmon across species and among populations.

Chinook salmon (*O. tshawytscha*) have wide life history diversity and many genetically distinct spawning populations that provide the opportunity for differences in average thermal experiences and adaptation (Bourret *et al.,* 2016). They are broadly distributed across a range of thermal environments from the warm Central Valley, California, across cool sub-arctic Alaska and back around the Pacific Rim to Japan, with populations declining and federally listed or assessed as endangered or threatened throughout their range (e.g. United States—ESA, 2022; Canada—COSEWIC, 2020). Central within their range, more than 50 distinct Chinook salmon populations return annually to migrate up the Fraser River, in British Columbia, Canada (Beacham *et al.,* 2002). Fraser River summer water temperature has increased by over 2°C since the 1950s, reaching over 22°C, (Foreman *et al.,* 2001; Patterson *et al.,* 2007) and is projected to continue increasing (Morrison *et al.,* 2002; Ferrari *et al.,* 2007; Grant *et al.,* 2019). Current knowledge suggests that fish are most vulnerable to warming temperatures as embryos/eggs and spawning adults (Pörtner and Farrell, 2008; Dahlke *et al.,* 2020), yet it remains unclear how elevated temperatures affect migrating adult Chinook salmon. In salmonids, physiology and morphology are strongly tied to thermal history, and adult sockeye (*O. nerka*) and embryo and juvenile Chinook salmon are locally adapted to their natal streams (Beacham and Murray, 1989; Beacham and Withler, 1991; Eliason *et al.,* 2011). Yet, we know remarkably little about the migrating adult life stage, nor how populations vary in thermal performance at this life stage and the mechanisms that underlie vulnerability, making conservation and management of Chinook salmon a challenge.

To determine the thermal sensitivity of migrating adult Chinook salmon, we examine the survival, metabolic capacities and recovery differences between two populations with distinct thermal histories. Survival is a clear indicator of success, especially for a semelparous species that has a single opportunity to spawn. Metabolism is also an important metric, because as water temperatures increase, so does aerobic cellular metabolism and therefore whole animal oxygen consumption rate (MO_2_, a proxy for metabolic rate) (Fry, 1971; Pörtner, 2001, 2010; Dillon *et al.,* 2010). But fish have a maximal capacity for aerobic metabolism, termed maximum metabolic rate (MMR) (Norin and Clark, 2016). As baseline maintenance metabolism or standard metabolic rate (SMR) increases with temperature, fish have a lower capacity to deliver oxygen to tissues to support aerobic activities such as swimming and migration (Farrell, 2016). This “capacity” is known as their “absolute aerobic scope” (AAS) and is the difference between their MMR and SMR (Fry, 1947; Farrell *et al.,* 2009; Pörtner, 2010; Eliason *et al.,* 2011; Schulte, 2015). Accordingly, AAS increases as a function of temperature until it is maximized, and fish are at their optimal thermal temperature (Topt_AAS_) and have a maximum capacity to perform aerobic activities, before declining at high temperatures (Farrell, 2016). As salmonids’ AAS is reduced due to increasing river temperatures, they may not be able to maintain the work needed to migrate upstream and complete spawning. In addition to aerobic swimming, migrating adult salmon must also use anaerobic burst swimming to negotiate hydraulic challenges, avoid predation, dig redds (nests), spawn and defend territories (Rand and Hinch, 1998; Healey *et al.,* 2003; Jain and Farrell, 2003; Berejikian *et al.,* 2007). Salmon can sustain aerobic swimming for extended periods, supporting migrations of hundreds of kilometers; however, they can only maintain anaerobic exercise for shorter durations. They must then restore homeostasis and metabolically recover by clearing lactate and restoring glycogen, high-energy phosphates, oxygen stores and osmoregulatory balance (Wood, 1991; Milligan, 1996; Kieffer, 2000; Jain and Farrell, 2003; Lee *et al.,* 2003a; Suski *et al.,* 2007; Raby *et al.,* 2015), a measurement termed “excess post exercise oxygen consumption” (EPOC) (Gaesser and Brooks, 1984). To complete upstream migration, salmon need to minimize both the duration and energetic costs of recovery (Claireaux *et al.,* 2000; Suski *et al.,* 2007; Eliason and Farrell, 2016). However, warming river temperatures may prolong recovery time (Prystay *et al.,* 2017; Kraskura *et al.,* 2021), which has clear fitness costs and could result in migration failure in Pacific salmon (Jain and Farrell, 2003; Lee *et al.,* 2003a; Eliason *et al.,* 2011; Burnett *et al.,* 2014b; Raby *et al.,* 2015).

Our objective was to compare the thermal performance of maturing and migrating adult Chinook salmon from the Fraser River. We compared two populations that experience different migration distances and challenges, including different thermal regimes: fall-run Coastal Chinook salmon from the Chilliwack population (125 km cooler migration), and summer-run Interior Chinook salmon from the Shuswap population (565 km warmer migration). We acclimated salmon to ambient conditions (12°C) and exposed salmon to acute warming temperatures either mimicking current, or future projected temperatures expected with climate change. We used intermittent respirometry to measure resting metabolic rate (RMR, a proxy to SMR), MMR following a chase and air exposure protocol, AAS and post-exercise recovery. We also assessed cardiac, red and white muscle enzyme activities (lactate dehydrogenase, citrate synthase), circulating blood plasma ion levels (K^+^, Cl^−^, Na^+^) and metabolite levels (lactate) to evaluate how populations differed in anaerobic and aerobic metabolic capacities and post-exercise recovery. Our hypothesis was that differences in physiological capacities would explain thermal sensitivities, matching historic riverine thermal conditions. We predicted that summer-run, interior Shuswap Chinook salmon would perform better at high temperatures, as indicated by higher survival rates, greater AAS breadth, a greater recovery capacity (e.g. lower lactate concentrations and plasma ion levels) and greater capacity for anaerobic and aerobic activity [e.g. greater AAS capacity, higher LDH and CS activities], compared with the fall-run, coastal Chilliwack Chinook salmon population. This work will help to elucidate mechanisms underlying intraspecific variability in thermal performance in ectotherms. In addition, by quantifying the thermal performance of adult Chinook salmon populations with different migration histories we can help inform conservation efforts of Chinook salmon across their geographic range.

**Figure 1:**
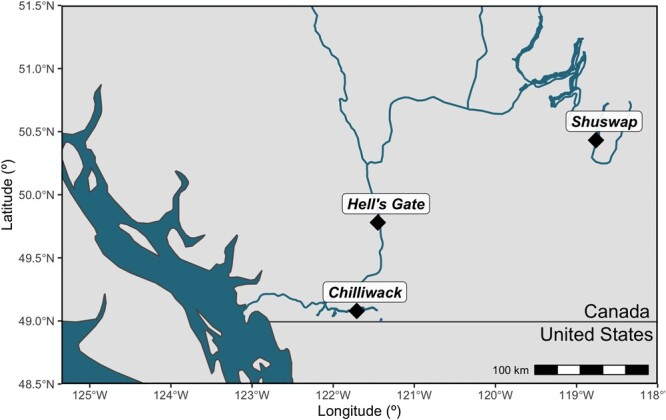
Map of Fraser River, British Columbia, Canada watershed. Spawning grounds for Chilliwack (Coastal) and Shuswap (Interior) Chinook salmon (*O. tshawytscha)* and the primary hydraulic challenge separating the Coastal and Interior populations (Hell’s Gate) are marked (black diamond).

## Methods

### Fish collection and holding

Chilliwack (N = 47, fork length (FL, snout to fork in tail in mm) = 632 ± 143 mm, mean ± SEM) and Shuswap (N = 38, FL = 673 ± 126 mm) Chinook salmon were collected *en route,* close to their respective spawning grounds: dip-netted at Chilliwack Hatchery (49.078550, −121.709216) October 7^th^ to 24^th^ 2019 and caught using seine-nets downstream of Mabel Lake (50.605414, −118.822505) September 17^th^ to 27^th^ 2019, respectively (Fig. 1). These populations experience different conditions during their spawning migrations in the Fraser River watershed. The “Coastal” Chilliwack Chinook salmon have a relatively easy migration (125 km). They are a fall-run and enter the Fraser River in September and arrive at Chilliwack Hatchery (220 m elevation) in October for spawning, encountering cooler temperatures (Daily mean = 15.5°C in 2019, Historic mean = 15°C from 1950–2018, Current maximum daily mean = 21.5°C) (Patterson *et al.,* 2007; Fraser River Ewatch, 2021). The “Interior” Shuswap Chinook salmon have a longer migration (565 km) and must pass through Hell’s Gate and the Fraser Canyon, a 200 km stretch of challenging swimming through fast waterflow, both posing considerable physical challenges, especially in warmer conditions (Martins *et al.,* 2012). Shuswap Chinook salmon are a summer-run and enter the Fraser River in July, soon enter the South Thompson River and migrate to their spawning grounds downstream of Mabel Lake (450 m elevation) in mid to late September and complete spawning by mid-October (Shearing, 2013). Because of their early entry, Shuswap Chinook salmon experience warmer conditions (Daily mean = 18.5°C in 2019, Historic mean = 18°C from 1950–2018, Current maximum daily mean = 22.8°C) in the lower Fraser River and South Thompson Rivers (Patterson *et al.,* 2007; Fraser River Ewatch, 2021).

After capture, the fish were transported by truck in a holding tank (2700 L, stocking density ≤ 15 fish per tank, > 90% air saturation) to the Cultus Lake Laboratory in Chilliwack, British Columbia, Canada (Fisheries and Oceans Canada). Fish were transferred to large outdoor holding tanks (5.3 m diam, 8000 L; stocking density ≤ 11 fish per tank) supplied with flow-through, sand-filtered and UV-sterilized freshwater from Cultus Lake. In each tank, air stones maintained oxygen > 90% air saturation and a water pump generated a circular current. The water temperature was maintained at 11–12°C by mixing warmer shallow water with colder deep lake water. Each tank had a transparent window to allow fish to maintain a natural diurnal cycle during the holding period. Fish were held for a minimum of 1 day and a maximum of 17 days prior to experimentation and were not fed. All experimental protocols were approved by the Animal Care Committee at the University of British Columbia (protocol #A17–0160).

### Intermittent flow respirometry

Five respirometers (54.5 or 98 L), custom-built from semi-transparent polyvinyl chloride tubes with a removable screw-on lid, held individual fish during the intermittent flow respirometry protocol for recordings of MO_2_. Fish were assigned to a respirometer according to size in order to maintain a 15:1 to 25:1 respirometer volume: animal mass. Water was continuously recirculated through the respirometer with a water pump (Eheim 1200 or Lifeguard Quiet One Pro 3000) and a flush circuit comprising a time-controlled flush pump (Lifeguard Quiet One Pro 5000, 45 L min^−1^ or Current USA E-Flux 3170, 48 L min^−1^) replenished the water and returned the oxygen levels to normoxia after each MO_2_ measurement. Oxygen (mg L^−1^) and temperature (°C) within the respirometers were recorded using a robust fiber optic oxygen sensor and temperature sensor (Pyroscience, Germany) placed in a PVC recirculating loop, which was connected to a FireSting optic O_2_ (and temperature) meter (Pyroscience, Germany). During a reading period, the flush pump was turned off and the decline in O_2_ levels due to the respiration of the fish was recorded. Chamber mixing was achieved by recirculation pumps as well as the ventilation and tail movements of the fish. The three experimental tanks holding the respirometers were sheltered with a tarp to minimize disturbance and the individual fish were oriented with their heads in the opaque caps, minimizin g visual disturbances.

Each experimental session started in the morning by transferring fish to an experimental thermal exposure tank (1.95 m diam, 1970 L, stocking density = 2 to 3 fish) maintained at 12°C. The temperature was then increased by 2°C h^−1^ until it reached the randomly assigned test temperature (12, 18, 21, or 24°C) and then held at the test temperature for 1 h (Supplementary Table S1). Fish were transferred using dip nets into an exercise tank (1.8 m diam, 2000 L) receiving a high flow-through of water maintained at the test temperature. Each fish underwent two exercise measurements: MMR_1h_, after 1 h of acute thermal exposure; and MMR_18h_, after 18 h of acute thermal exposure. MMR_1h_ occurred 13:00–14:30 and the MMR_18h_ occurred 8:00–9:00 the following day. To determine MMR, the fish were exercised to exhaustion by manually “chasing” the fish to elicit swimming or burst swimming for 3 min, by lightly following or touching the individuals’ tail, followed by 1 min air exposure in a dip net (Gale *et al.,* 2014; Little *et al.,* 2020a). Following air exposure, fish were immediately (within 120 s) transferred into respirometers submersed in flow-through experimental tanks (181 cm diam, 42 cm depth) maintained at the test temperature (via mixture of heated water and cold lake water) to measure MO_2_ following the chase protocol and during the subsequent recovery over the following 18–20 h during which RMR was determined (more details below). After 18–20 h at the exposure temperature, the fish were removed from the respirometer and underwent another identical chase exercise protocol as described above, and then immediately returned to the respirometer to measure immediate MO_2_ (MMR_18h_). The first MO_2_ measurement following MMR_1h_ and MMR_18h_ consisted of a 4–6 min closed DO measurement during which the flush pump was turned off allowing assessments of MO_2_, and then by flushing the respirometer to fully reoxygenate the respirometer. This was followed by automated 10- or 15-min MO_2_ cycles, comprising 6–9 min flushing periods to fully reoxygenate the respirometer followed by 4–6 min flush-off periods until the removal of the fish. The timing of the MO_2_ cycles was adjusted to ensure that the O_2_ remained above 75% air saturation. Test temperatures were selected to reflect current Fraser River temperatures on the lower (12°C) and upper-range (18°C) and future projected temperatures (21, 24°C) (Fraser River Ewatch, 2021). The overall brief temperature duration (i.e. 1 h of temperature exposure and 18 h of temperature exposure) was specifically chosen to mimic an acute, short-term ecologically relevant heat stress event (Rodnick *et al.,* 2004; Hague *et al.,* 2011).

Recovery occurs over multiple timescales, including partial, but rapid, initial recovery (within minutes to hours, important for repeat bouts of maximum swim performance) (Farrell *et al.,* 1998; Jain *et al.,* 1998; Jain and Farrell, 2003; Lee *et al.,* 2003a) and full recovery (up to 16 hours, important for repairing cellular damage and restoring metabolites, SMR, resting heart rate) (Milligan, 1996; Zhang *et al.,* 2018). We sampled fish after 1 h of recovery when the initial rapid recovery phase was expected to be complete and under optimal conditions fish would be able to resume swimming (e.g. Eliason *et al.,* 2013b), however full recovery would not be expected. Following the MMR_18h_ measurement and 1 h recovery period, the fish were removed from the respirometer and euthanized by a blunt cranial blow followed by severing the spinal cord with a scalpel. Fish exhibiting signs of morbidity throughout the experiment (e.g. loss of equilibrium, gasping at surface of exposure tank) were immediately euthanized. Trials occurred over 26 days and systems were cleaned between each population using a diluted Virkon disinfectant. Probe calibration occurred weekly using two-point calibrations (aerated water for 100%, sodium sulfite for 0%).

### Terminal sampling and body morphometrics

Each fish was measured for mass (to the nearest gram) and fork length (to the nearest mm). A caudal blood sample (~3 ml) was collected (21 G needle, lithium heparinized BD Vacutainer, BD, Franklin Lake, NJ, USA) and immediately placed on ice for a maximum of one hour. Hematocrit was measured in duplicate and the remaining blood was centrifuged at 1200 g for 5 min to separate the blood plasma, which was immediately flash frozen in liquid nitrogen and stored at −80°C for later analyses. Heart (bisected across the valve to the apex) and muscle tissues (two samples ~ 0.5 cm thick containing both red and white muscle posterior to the dorsal fin) were freeze clamped in liquid nitrogen and stored at −80°C for later analyses. Organ masses were recorded for ventricle, gonads, liver and spleen and the adipose fin was marked as present (i.e. wild fish) or absent (i.e. hatchery origin fish). The gonadal somatic index (GSI), relative ventricular mass (RVM), splenosomatic index (SSI), hepatosomatic index (HSI) were calculated by dividing organ mass (gonads, ventricle, spleen, or liver) by the fish total body mass ^*^ 100.

### Blood and tissue analyses

Plasma and tissue metrics reveal possible mechanisms underlying the performance of individuals. Potassium and sodium were analyzed using an XP Five-channel Flame Photometer (BWB Technologies, UK), chloride using a Chlorocheck Digital Chloridomter (EliTech Group, France) and osmolality using a 3200 Osmometer (Advanced Instruments, USA). Elevated or depressed ions indicate an ionic imbalance and, particularly following exhaustive exercise, can disrupt muscle contraction and inhibit swimming (Wood, 1991; Holk and Lykkeboe, 1998). Plasma lactate and glucose were measured using a 2300 Stat Plus Glucose and L-Lactate analyzer (YSI, USA) according to established methods (Farrell *et al.,* 2001). Glucose is a finite energy reserve that fuels metabolism and is mobilized during stressful events (Kubokawa *et al.,* 1999). Hormones were run in a FLUOstar Omega multi-mode microplate reader (BMG Labtech, USA). Cortisol was analyzed via Cortisol ELISA kits (Neogen, USA) and read for absorbance at 650 nm, followed by the addition of 50 μl 1 N HCl and measured at 450 nm. Cortisol has many functions and elevated levels have been linked to impaired performance in Pacific salmon (Milligan, 1996). Testosterone and 17B-estradiol were extracted from plasma using diethyl ether and quantified with ELISA kits (Neogen, USA) according to manufacturer instructions. Reproductive hormones promote sexual maturation and indicate the reproductive status of individuals (Idler *et al.,* 1961). All plasma samples were run in duplicate.

Lactate results from anaerobic glycolysis and indicates physiological recovery status from anaerobic exercise (Milligan, 1996). To measure tissue lactate, frozen ventricle and white muscle samples were ground under liquid nitrogen using a mortar and pestle and weighed (~20 mg), treated with ice-cold 8% HClO_4_ and sonicated on ice with three 5 s bursts. The homogenate was centrifuged at 10000 g for 10 min at 4°C and the supernatant was neutralized using 3 M K_2_CO_3_, centrifuged again at 10000 g for 10 min at 4°C and extracts were aliquoted (~400 μl) and stored at −80°C until analyses. Samples were assayed in triplicate on a FLUOstar Omega Microplate reader with a lactate standard curve to measure the concentration of lactate using LDH to catalyze the oxidation of lactate with the reduction of NAD^+^ at 340 nm (Richards *et al.,* 2002).

If oxygen supply is limited during exercise or post-exercise recovery, aerobic and anaerobic metabolic proxies indicate both the ability and capacity to sustain performance (Brett, 1964). The capacity for aerobic (e.g. CS) and anaerobic metabolism (e.g. LDH) in tissues with high ATP demand (e.g. heart, red muscle, white muscle) might be locally adapted to thermal conditions and enzyme activity levels measured at different assay temperatures across groups (sex, populations, species) could reveal differences in thermal adaptation (Little *et al.,* 2020b). Enzyme activities (CS and LDH) were measured from the ventricle, white and red muscle homogenates to determine the thermal performance of these tissues across 8, 12, 18, 24 or 25 and 28°C using established methods (Moyes *et al.,* 1997; Martínez *et al.,* 2006; Little *et al.,* 2020b). Frozen tissues were sliced, weighed (~25 mg) and homogenized in buffer (0.1% Triton, 50 mmol l^−1^ HEPES, 1 mmol L^−1^ EDTA, pH 7.4) with 0.5 mm zirconium oxide beads in a bead beater (Fisherbrand Bead Mill 24 Homogenizer) kept at 4°C for two 6 m s^−1^ 30 s cycles with 1 min on ice in-between. Aliquots were separated (~300 μl each) and stored at −80°C until analyses for LDH and CS. All samples were read in triplicate on a FLUOstar Omega multi-mode microplate reader (BMG Labtech, Germany) at 340 nm to measure the disappearance of NADH for LDH activity, or 412nm to measure the production of 5-thio-2-nitrobenzoic acid, a proxy for CS activity. Activity levels were calculated with an extinction coefficient of 6.22 and 13.6 mmol^−1^ cm^−1^ for LDH and CS, respectively. Absorbance readings were normalized using the Pathlength sensor.

### Data and statistical analyses

Fish with low hematocrit (<20%) were excluded from the study (4 fish total). The MO_2_ data were analyzed and visualized in RStudio (RStudio Team, 2020) using custom code (Kraskura, 2022). The mass-specific MO_2_ (expressed in mg O_2_ kg^−1^ min^−1^) was calculated from the change in the concentration of O_2_ over time (∆O_2,_ i.e. the slope of a fitted regression line over the course of each measurement cycle) in the sealed respirometer using MO_2_ = (∆O_2_ *(v_R_-v_F_))/m, where v_R_ is respirometer volume, v_F_ is mass of the fish (L, assuming 1 kg = 1 L) and m is the fish mass (kg).

MMR was calculated in three ways: MMR_1h_, MMR_18h_ and MMR_OVERALL_ (see below). MMR_1h_ and MMR_18h_ were calculated from the first measurement cycle following the exhaustive exercise protocol using a sliding window analysis (90 s minimum). Specifically, each ≥90 s sliding window began at the start of the measurement period and moved in 1 s increments across the measurement cycle, and the steepest ∆O_2_ with an R^2^ > 0.9 was used as MMR (Little *et al.,* 2020a). Depending on life stage and behavior, exhaustive exercise does not always evoke the highest MO_2_, which is what defines MMR (Raby *et al.,* 2020) so we also estimated MMR_OVERALL_ by choosing the maximum MO_2_ value measured during the experiment for each fish with more than 60 overnight recovery MO_2_ values. Because there was a minimal effect of time exposed to temperature, we used the MMR_OVERALL_ in our primary analyses to best estimate maximum MO_2_.

RMR typically occurred during nighttime and was calculated as the lowest 10% quantile of all validated MO_2_ measurements with R^2^ > 0.85. RMR was calculated for individuals that had at least 60 validated MO_2_ measurements. All regressions were visually assessed to ensure slopes were linear, negative and with no artificial irregularities. Fish that died during RMR measurements were not included in the RMR estimate. AAS values were calculated as the MMR_OVERALL_ – RMR for each individual, and factorial aerobic scope (FAS) values were calculated as MMR_OVERALL_/RMR. In cases where fish did not survive the experiment (from the beginning of the acute 1 h thermal exposure to the end of the MMR_18h_ recovery period), an individual was considered a mortality and AAS and FAS were treated as values of 0 because a dead fish is assumed to have zero aerobic scope. Further, it is clear from moribund fish that aerobic capacity is severely diminished. While it is rare to include mortalities in estimates of aerobic scope, these fish died during the experimental procedures (i.e. during the respirometry measurements or 1 h thermal exposure period before the respirometry trial began) and thus are part of the full, complete dataset. We also present results from survivors only but point out that this method could be misleading and may greatly overestimate the aerobic capacities of these populations.

Allometric scaling of metabolism was considered because of the large range in body mass, 1.4 to 7.2 kg. Mixed models were used to test the significance of the following main predictor variables: population and test temperature (with and without the interaction) and log_10_(body mass). Model selection criteria (Bayesian Information Criterion, BIC) were used to determine the best-fit model for each performance metric and confirmed allometric scaling of metabolism. The significance of each main effect was tested using ANOVA. For visualization purposes, the values are presented as a mass-specific (mg O_2_ kg^−1^ min^−1^) adjusted to represent a 3.5 kg fish using metabolic scaling coefficients estimated by mixed models (scaling exponent of 0.67 for RMR and 0.58 for MMR_1h_, MMR_18h_ and MMR_OVERALL_).

Background microbial respiration rates were measured for 30 min before and after each experiment for each respirometer. However, the microbial respiration was determined to be negligible and therefore not incorporated into analyses.

We measured short-term recovery with three estimates: (1) Percent of AAS (%AAS), calculated as the MO_2_ recovered as a function of the AAS following each exercise calculated at time points 0, 10, 20, 30, 40 and 50 min; (2) percent of MMR (%MMR), calculated as the MO_2_ value as a function of the MMR_1h_ or MMR_18h_ following the respective exhaustive exercise, calculated at time points 0, 10, 20, 30, 40 and 50 min; and (3) the time to recover to 50% of respective MMR (recMMR_50_) (Kraskura *et al.,* 2021). Individuals without a distinct measurement between 48 and 52% of MMR were excluded from recMMR_50_ analyses. EPOC was analyzed by smoothing MO_2_ measurements using a cubic smoothing spline function (smooth.spline, R package ‘stats’) for overnight recovery measurements starting at MMR_1h_. EPOC was calculated as the area integrated beneath the curve minus the area of the integrated RMR, with values pooled and calculated into the first five hourly time blocks. Fish that did not have > 60 measurements or did not complete EPOC (return to RMR; one individual) were excluded from this analysis.

Temperature coefficients (Q_10_) for RMR values were calculated based on temperature treatment group means for each population using the equation,

$$ Q10={\frac{RX}{R12}}^{\left(\frac{10}{TX-T12}\right)} $$

where R12 and RX are the RMR values measured at corresponding temperature T12 (12**°**C) and TX (18°C, 21°C or 24**°**C).

The optimal temperature for AAS (Topt_AAS_) was measured as the maximum AAS values based on the polynomial model calculated for AAS and the upper T_pejus_ (°C) was defined as the maximum temperature at which AAS remained above 80% of the maximum AAS.

All data were analyzed for statistics using RStudio version 1.2.1335 (RStudio Team, 2020). Statistical significance was accepted at P < 0.05. Values are presented as mean ± standard error of mean (SEM) unless otherwise stated. Values were assessed for normality using residual plots and quantile-quantile plots and log_10_-transformed if necessary to fit normality assumptions. All data were measured for homoscedasticity using Levene’s Test. For survival rates, a binomial two-parameter log-logistic function was fit and a likelihood ratio test compared the model with the fixed effect (population) to a null model. Body mass differences across test temperatures within and across populations were assessed using a two-way ANOVA. The effects of sex and population on body metrics (GSI, RVM, SSI, HSI) were assessed using a two-way ANOVA with sex and population as factors. MMR_1h_, MMR_18h_, MMR_OVERALL_, RMR, AAS, FAS, recMMR_50_, plasma variables and tissue lactate were modeled for interactive effects of temperature and population and analyzed using two-way ANOVA’s and significant main effects or interactions were further explored using a Tukey’s HSD post hoc test (R package ‘emmeans’). When an interaction was not significant, the interaction was dropped and the model was re-run to test for main effects. To fit parametric test assumptions, recMMR_50_ values were log10-transformed. The AAS and FAS were also modeled to a second order polynomial regression for the entire dataset (mortalities included as 0’s). To determine the effect of time exposed to test temperature on post-chase MMR (MMR_1h_ vs MMR_18h_), a linear mixed-effects model fit by Maximum Likelihood was used. The main effects were exposure time (1 *vs*. 18 h), population and temperature treatment as categorical predictors, and the random effect was fish ID because individual fish were measured for MMR at both time points. The significance of each main effect was tested using a type II two-way ANOVA (R package ‘nlme’; Pinheiro *et al.,* 2022).

Recovery data (%AAS, %MMR, hourly EPOC and cumulative EPOC) were non-independent across time and were analyzed using repeated measures ANOVA. We used linear mixed models to account for individual-specific trends, with individual fish as a random effect to account for repeated measures across each timepoint (R package ‘lme4’) (Bates *et al.,* 2015). For the short-term recovery (1 h) following exhaustive exercises MMR_1h_ and MMR_18h_, both %AAS and %MMR values were pooled in 10 min blocks for averages within each temperature treatment for MMR_1h_ and MMR_18h_. The timepoints (10 min intervals), temperature treatment and population and their interactions were all included as fixed effects. The best fit model as determined using BIC did not include the interaction between population and any covariates. The significance of each fixed effect was then measured using a two-way ANOVA (type III). Hourly and cumulative EPOC were log10-transformed to comply with parametric test assumptions.

Kinetic enzyme activities were analyzed for differences using mixed effect models with effect terms: population, assay temperature and their interaction. Fish ID was used as a cluster variable to account for repeated measures across different assay temperatures using a two-way ANOVA (type III).

## Results

### Body morphometrics

Body mass was approximately the same across all treatments in Chilliwack (3.31 ± 0.25 kg, N = 47) and Shuswap (3.56 ± 0.21 kg, N = 38) Chinook salmon (pop^*^temp: F_3_ = 0.191, P = 0.902, Supplementary Table S1). Though we attempted to control for reproductive status by collecting all fish near spawning grounds, there was an interactive effect of population and sex on GSI (F_1_ = 19.410, P < 0.0001, N = 85). Sexual maturity, as determined by greater GSI, varied between populations. Female Chilliwack Chinook salmon had a lower GSI compared with female Shuswap salmon (19.74 ± 1.37% [N = 5] *vs*. 23.47 ± 1.13% [N = 12] respectively; P = 0.0013), whereas male Chilliwack salmon had a greater GSI than male Shuswap salmon (7.07 ± 0.29%; [N = 42] *vs.* 5.25 ± 0.20% [N = 26], respectively; P = 0.0017).

Males had a greater RVM than females in both Chilliwack and Shuswap Chinook salmon (males 0.20 ± 0.00% and 0.20 ± 0.01% vs. females 0.17 ± 0.01 and 0.17 ± 0.00%, respectively, across populations; F_1_ = 10.856, P = 0.001, N = 85). Both population and sex independently influenced SSI, where male Chilliwack salmon had a 32% greater mean SSI compared with male Shuswap salmon (0.21 ± 0.01 (N = 42) *vs*. 0.16 ± 0.01% (N = 26)), respectively, but there were no major difference in SSI between females (Chilliwack: 0.11 + 0.01% (N = 5) *vs*. Shuswap: 0.12 + 0.01% (N = 12)) (sex: F_1_ = 15.393, P < 0.001; pop: F_1_ = 8.218, P = 0.005). In contrast, there were no differences in HSI between populations or sex (Chilliwack Chinook salmon: 1.79 ± 0.08% *vs*. Shuswap Chinook salmon: 1.50 ± 0.15%; pop: F_1_ = 2.152, P = 0.146; sex: F_1_ = 0.522, P = 0.472).

### Effects of warming on survival

**Figure 2:**
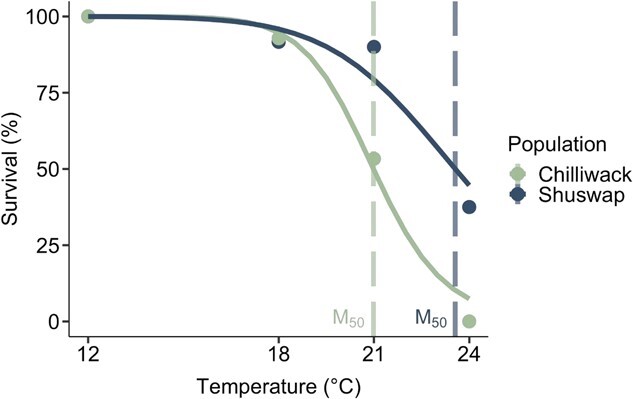
Chinook salmon population survival across test temperatures. The percent of surviving individuals from the Chilliwack (green symbols; *N* = 47) and Shuswap (blue symbols; *N* = 38) Chinook salmon (*O. tshawytscha*) populations are plotted as data points fitted with a two-parameter log-logistic function. Vertical dashed line indicates M_50_, the temperature at which 50% mortality was predicted to occur in each population (M_50_ = 21°C, 95% CI [20.0, 22.0°C] Chilliwack, 23.6°C, 95% CI [21.2, 25.9°C] Shuswap).

Both populations suffered mortalities throughout the experiment (i.e. starting at the acute 1 h temperature exposure before respirometry through to 1 h post MMR_18h_) at 18, 21 and 24**°**C. The mortality rates at 18**°**C were similar but low, and differences in survivorship became evident at 21**°**C, where 47% of Chilliwack Chinook salmon and 10% of Shuswap salmon died (Fig. 2, Supplementary Table S2). Differences in survivorship were stark at 24**°**C, with 100% mortality in Chilliwack Chinook salmon and 63% mortality in Shuswap Chinook salmon (Supplementary Table S1). There were not enough female Chinook salmon in either population to evaluate statistical differences in sex survival rates.

Survival rate decreased in both populations with increasing temperature (Fig. 2). Population as a fixed effect on the survival rate model had a marginally better fit than the null model (χ2_4_ = 4.9452, P = 0.0844; Fig. 2). Fifty percent of mortalities (M_50_) occurred at lower temperatures for Chilliwack Chinook salmon, with an M_50_ of 21.0°C, 95% CI [20.0, 22.0°C] compared with an M_50_ of 23.6°C, 95% CI [21.2, 25.9°C] for Shuswap Chinook salmon (Fig. 2). Mortality occurred following the first exhaustive exercise event (MMR_1h_) resulting in fewer MMR_18h_ measurements at several temperatures (Chilliwack: N = 5 mortalities during recovery at 21°C, N = 4 at 24°C; Shuswap: N = 1 at 18°C and N = 3 at 24°C). Differences in sample sizes were also due to the inability to use some MMR values based on requirements described above.

### Metabolic performances

**Figure 3:**
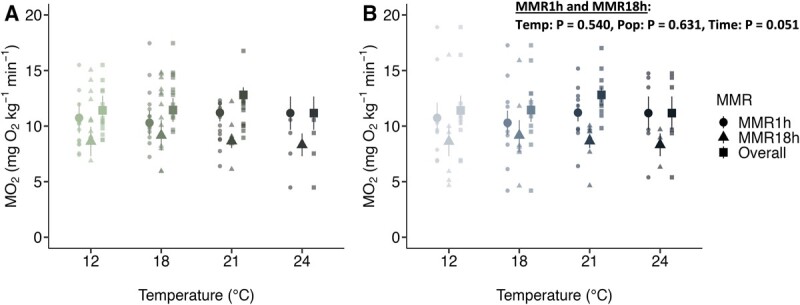
MMR from three separate measurements. The MMR during 1 h exposure (MMR_1h_), 18 h exposure (MMR_18h_) and calculated from maximum values including during overnight recovery (MMR_OVERALL_) in (A) Chilliwack (green symbols) and (B) Shuswap (blue symbols) Chinook salmon (*O. tshawytscha*) acutely exposed to 12, 18, 21, or 24°C. Large data points are mean MMR values ± SEM and small data points are individual MMR values. Values are corrected using the metabolic scaling coefficient of 0.58. Note that there is no data for MMR_18h_ for Chilliwack fish at 24°C because of mortalities. Statistical results from repeated measures two-way ANOVA accounting for MMR_1h_ and MMR_18h_ values are presented with variables: test temperature (Temp), population (Pop), MMR time (Time), fish id (ID)) in panel B (MMR ~ Temp + Pop + Time, random = ~ 1|ID).

There were no effects of test temperature or population, when comparing MMR_1h_ and MMR_18h_ values, though there was an effect of time exposed to test temperature (temp: Χ^2^_3_ = 2.159, P = 0.540; Pop: Χ^2^_1_ = 0.230, P = 0.631; time: X^2^_1_ = 3.815, P = 0.051; Fig. 3, Supplementary Table S3). Some fish (39%; N = 14 for Chilliwack and N = 10 for Shuswap of 61 statistically eligible fish) experienced their highest MO_2_ values during their overnight recovery, and for those fish, values were 26% and 40% higher than MMR_1h_ for Chilliwack and Shuswap Chinook salmon, respectively (Fig. 3). Therefore, the remainder of the results presented use MMR_OVERALL_ values.

**Figure 4:**
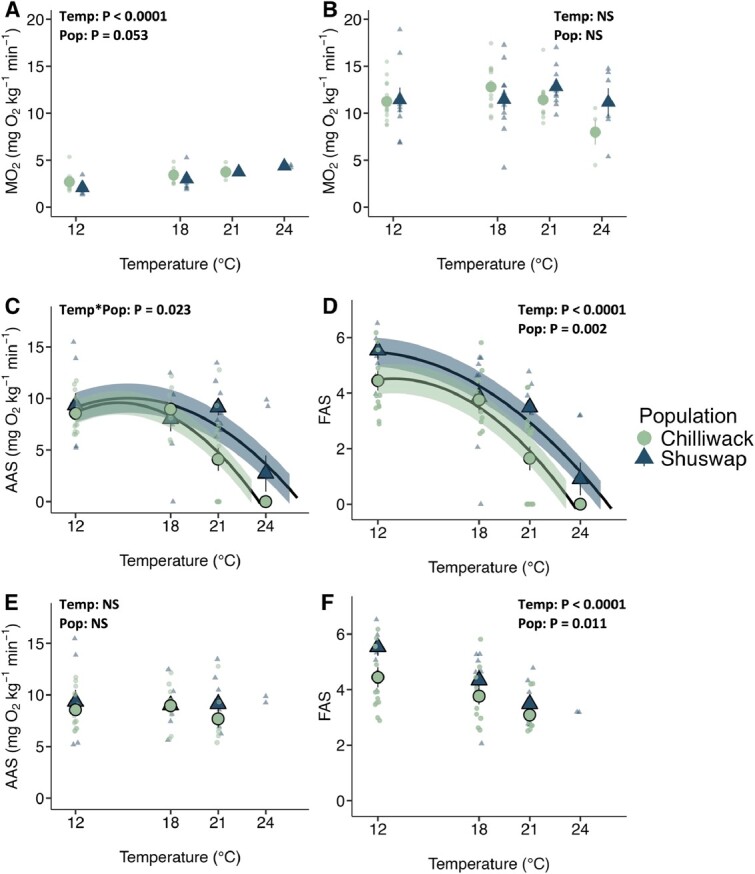
Effects of warming on metabolic rates in Chilliwack Chinook salmon (*O. tshawytscha*) (green circles) and Shuswap Chinook salmon (blue triangles) acclimated to 12°C and tested at different temperatures (18, 21, 24°C). (A) Resting metabolic rate, (B) MMR (MMR_OVERALL_), (C) AAS of all fish (mortalities included as 0), (D) FAS of all fish (mortalities included as 0), (E) AAS of surviving fish, (F) FAS of surviving fish. Significant effects (test temperature (Temp), population (Pop)) denote statistical results for RMR, MMR_OVERALL_, AAS and FAS. A second order polynomial regression is modeled as (AAS ~ temp + I(Temp^2^) and (FAS ~ Temp + I(Temp^2^) with SEM shaded for (C) and (D)). Values are corrected to a common body mass of 3.5 kg using the metabolic scaling coefficient of 0.58 for MMR_OVERALL_ (mg O_2_ kg ^−1^ min^−1^), 0.67 for RMR (mg O_2_ kg ^−1^ min^−1^), AAS as MMR_OVERALL_ -RMR (mg O_2_ kg ^−1^ min^−1^), FAS as MMR_OVERALL_/RMR (unit-less) and expressed as mean ± SEM. Individual data points represent values for individual fish. Mortalities in (C) and (D) occurred during the experiment and are indicative of zero scope (Chilliwack: 12°C [N = 0], 18°C [0], 21°C [7], 24°C [4]; Shuswap: 12°C [N = 0], 18°C [1], 21°C [0], 24°C [5]). Note that there are no RMR data at 24°C for Chilliwack Chinook salmon due to mortality, and SEM is too small to be seen for some values. Also note there are no mean or SEM for 24°C in (E) and (F) due to N = 2.

At the acclimation temperature (12°C), there are minimal differences in metabolic rates (RMR, MMR_OVERALL_, AAS, FAS) between populations (Fig. 4). Chilliwack Chinook salmon and Shuswap Chinook salmon had similar RMR values (P = 0.053, 2.70 ± 0.24 *vs*. 2.06 ± 0.21 mg kg^−1^ h^−1^, respectively) and there were no differences in MMR_OVERALL_ (P = 0.545, 11.26 ± 0.55 *vs*. 11.41 ± 1.33 mg kg^−1^ h^−1^, respectively; Fig. 4, Supplementary Table S3). The resulting AAS also showed no difference between Chilliwack and Shuswap Chinook salmon populations at 12°C (P = 0.565, 8.56 ± 0.51 vs. 9.35 ± 1.14 mg kg^−1^ h^−1^, respectively), though Shuswap Chinook salmon had a greater FAS than Chilliwack Chinook salmon at 12°C (*P* = 0.002, 4.45 ± 0.36 vs. 5.54 ± 0.33, respectively; Fig. 4, Supplementary Table S3).

Temperature effects were apparent across several metrics. RMR increased with increasing test temperatures (18, 21 and 24°C) in both Chilliwack and Shuswap Chinook salmon (F_3_ = 12.886, *P* < 0.0001; Fig. 4, Supplementary Fig. S1, Supplementary Table S3). RMR values were not reported for Chilliwack Chinook salmon at 24°C because of high mortality. Shuswap Chinook salmon displayed a higher thermal sensitivity relative to Chilliwack Chinook salmon (RMR Q10_12–18_ = 1.86 vs. 1.49, Q10_12–21_ = 1.93 vs. 1.44, respectively, and Q10_12–24_ = 1.87 (Shuswap only)). However, neither test temperature nor population origin affected MMR_OVERALL_ in either population (temp: F_3_ = 1.938, P = 0.131; pop: F_1_ = 0.371, P = 0.545), with mean values ranging from 7.99 to 12.80 mg O_2_ kg^−1^ min^−1^ in Chilliwack Chinook salmon and 11.17 to 12.81 mg O_2_ kg^−1^ min^−1^ in Shuswap Chinook salmon (Fig. 4, Supplementary Table S3).

**Figure 5:**
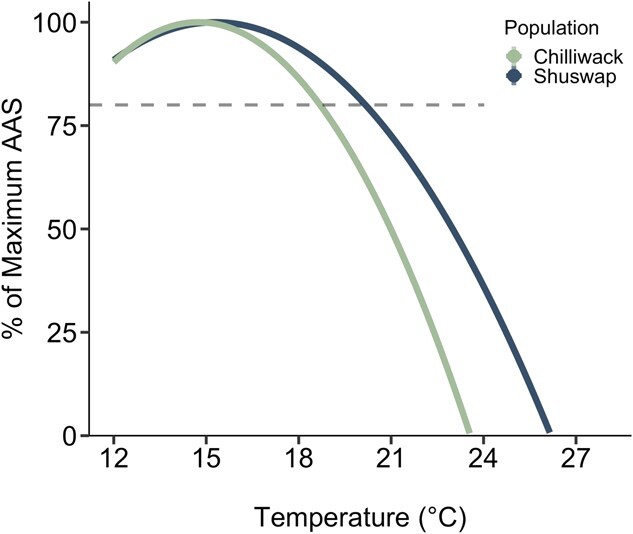
Predicted river temperature thresholds for Fraser River Chinook salmon (*O. tshawytscha*) populations. AAS is modeled as percent of maximum AAS for Chilliwack (green) and Shuswap (blue) Chinook salmon across temperatures based on a second order polynomial regression (AAS ~ Temp + I(Temp^2^)). Pacific salmon are hypothesized to need 80% of maximum AAS (horizontal dashed line) to successfully spawn, corresponding to the upper T_pejus_ at 18.70 ± 2.96°C (SD) (Chilliwack Chinook salmon) and 20.15 ± 3.70°C (Shuswap Chinook salmon).

Aerobic scope was assessed in two ways: (1) incorporating fish that died during the experimental period as zero aerobic scope values (Fig. 4C, D) only from survivors (Fig. 4E, F). When incorporating fish that died, AAS varied among temperature treatments in Chilliwack and Shuswap Chinook salmon (F_3_ = 16.096, P < 0.0001; Fig. 4C, Supplementary Table S3). For Chilliwack Chinook salmon, AAS was both greatest and unchanged between 12 and 18°C (8.56 ± 0.51 and 8.96 ± 0.58 mg O_2_ kg^−1^ min^−1^, respectively). However, due to increasing RMR and relatively unchanged MMR_OVERALL_ with increasing temperature, AAS declined at 21 and 24°C in both populations, with no scope remaining in Chilliwack Chinook salmon at 24°C because of mortalities, and very limited scope in Shuswap Chinook salmon at 24°C (2.73 ± 1.76 mg O_2_ kg^−1^ min^−1^). The thermal range for Topt_AAS_ and upper T_pejus_ was 14.75–18.70 and 15.30–20.15°C reaching 9.6 and 10.0 mg O_2_ kg^−1^ min^−1^ in Chilliwack and Shuswap Chinook salmon respectively (Fig. 4C; Fig. 5). Test temperature also affected FAS (F_3_ = 32.343, *P* < 0.001; Fig. 4D, Supplementary Table S3). In Chilliwack Chinook, FAS decreased from 12°C to 18°C (4.45 ± 0.36 and 3.76 ± 0.30, respectively) and fish had more than two times the amount of FAS available than at 21°C (1.65 ± 0.43). Shuswap Chinook salmon had the highest FAS overall at 12°C (5.54 ± 0.33) but more rapidly declined with increasing temperatures, dropping by 30% at 18°C (3.86 ± 0.59) and nearly 40% by 21°C (3.49 ± 0.24). When aerobic scopes were assessed for surviving fish only (Fig. 4E, F; Supplementary Table S3), there was no effect of temperature (F_2_ = 0.356, P = 0.702) or population (F_1_ = 1.500, P = 0.226) on AAS. However, there were significant effects of temperature (F_2_ = 12.871, P < 0.0001)) and population (F_1_ = 6.968, P = 0.011) on FAS (Fig. 4F).

### Post-exercise recovery

**Figure 6:**
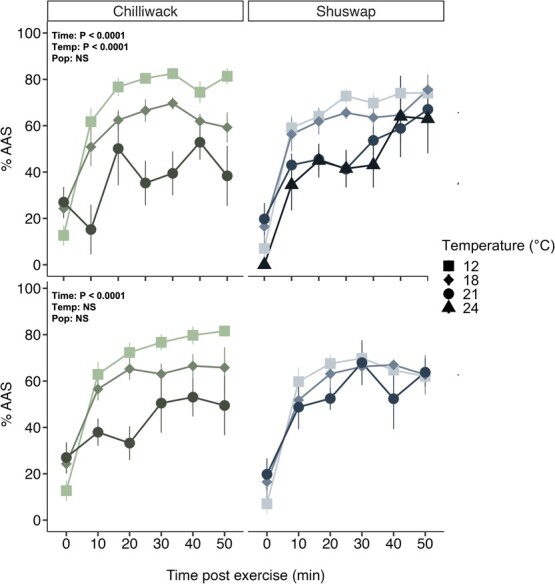
Short-term recovery following exhaustive exercise in Chinook salmon (*O. tshawytscha*). Short-term recovery measured as the MO_2_ recovered as a function of AAS during the 50 min of recovery following (A) MMR_1h_ and (B) MMR_18h_ for Chilliwack (green symbols; first column) and Shuswap (blue symbols; second column) Chinook salmon acclimated to 12°C and tested at 12 (square), 18 (diamond), 21 (circle) and 24°C (triangle). Values are pooled every 10 min as mean percent of AAS ± SEM. Significant effect of each effect term (timepoint (Time), test temperature (Temp), population (Pop)) (ANOVA) denote statistical results for recovery following each exhaustive exercise (MMR_1h_ or MMR_18h_). Note that there are no recovery values for 24°C Chilliwack Chinook salmon at either chase timepoint (MMR_1h_, MMR_18h_) because of mortality following the first exhaustive exercise recovery period and no data for 24°C Shuswap Chinook salmon at the 18 h exhaustive exercise because of low sample size (*N* = 2). Note that percent AAS at 0 min does not begin at 0% because AAS is calculated based on MMR_OVERALL_.

Short-term (1 h) recovery after exercise was never complete, even at 12°C (Fig. 6). Both the rapid (1 h) and acute (18 h) temperature exposure times resulted in drastic performance differences in both populations, most noticeably at the extreme upper temperatures (Fig. 6; Supplementary Fig. S2). Test temperature impacted %AAS after MMR_1h_ (P < 0.0001) but not MMR_18h_ (P = 0.263; Fig. 6). Timepoint affected %AAS after MMR_1h_ and MMR_18h_ (P < 0.0001) whereas there was no effect of population on %AAS after either respective chase (P = 0.132, 0.129; Fig. 6). There was also a significant two-way interaction between the timepoint and temperature treatment following MMR_1h_ (time*temp: χ2_1_ = 11.807, P < 0.001; Supplementary Fig. S2). In both populations and at both exposure times, fish held at the lowest test temperatures (12, 18°C) recovered more rapidly, with more than 50% AAS within the first 10 min. In Chilliwack Chinook salmon, fish held at the higher test temperatures (21°C, 24°C) recovered more slowly and did not return to 50% AAS within the first 50 min of recovery, whereas the Shuswap Chinook salmon tested at the higher test temperatures recovered to 50% AAS after both chases within the 50 min recovery (Fig. 6). The recovery time required to reach 50% of the MMR following the first exhaustive chase MMR_1h_ displayed similar patterns as described here for %AAS (Supplementary Fig. S2).

Intermediate (5 h) recovery from MMR_1h_ as measured by EPOC acquired each h over five h (Supplementary Fig. S3) varied due to a two-way interaction between hourly timepoints and test temperature (time*temp: χ^2^_12_ = 41.401, P < 0.01), though there was no effect of population (χ^2^_1_ = 0.353, P = 0.552).

### Blood chemistry & hormones

**Figure 7:**
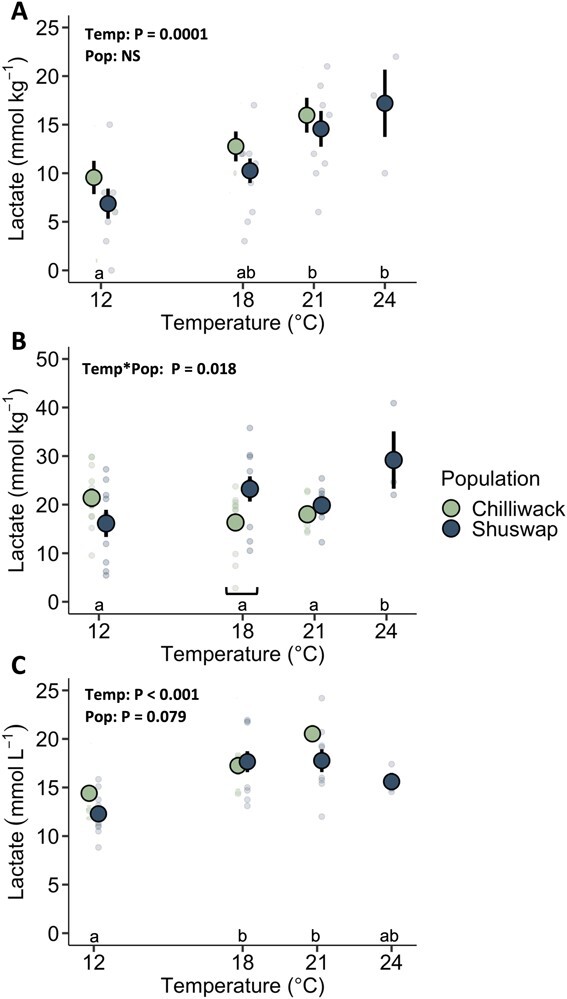
Lactate concentration in Chinook salmon (*O. tshawytscha*) (A) cardiac tissue, (B) white muscle and (C) plasma after 1 h recovery. Concentration (cardiac and white muscle: mmol kg^−1^; plasma: mmol L^−1^) measured from individuals acclimated to 12°C and tested at 12, 18, 21 and 24°C is presented as mean ± SEM. Faded individual data points represent values from individual fish, with associated population color. Significant two-way interaction (test temperature (Temp) * population (Pop)) (ANOVA; Table 1; Supplementary Table S4) or effect terms independently (ANOVA; Supplementary Table S4) denote statistical results for each tissue, where letters represent a significant difference between test temperatures and bar represents a significant difference between populations at a test temperature.

In general, blood values sampled after 1 h of recovery following exhaustive exercise (MMR_18h_) varied across populations and temperature treatments. There were significant effects of both population and temperature on plasma lactate at the 1 h recovery timepoint post MMR_18h_, with increases in lactate with increasing temperature from 14.40 to 20.52 mmol L^−1^ in Chilliwack Chinook salmon and 12.29 to 17.77 mmol L^−1^ in Shuswap Chinook salmon (pop: F_1_ = 3.189, *P* = 0.079; temp: F_3_ = 12.761, *P* < 0.001; Table 1, Fig. 7C). Male salmon testosterone levels significantly decreased with elevated temperature across populations, declining by 49% and 76% from 12 to 21°C in Chilliwack and Shuswap Chinook salmon, respectively (pop*temp: F_2_ = 4.278, P = 0.020; Table 1). There were also significant effects of population on estradiol in males, with estradiol concentrations 30% higher in Chilliwack Chinook salmon than Shuswap Chinook salmon at 12°C (pop: F_1_ = 26.022, *P* < 0.001; Table 1). Male Shuswap Chinook salmon had lower cortisol levels compared with male Chilliwack Chinook salmon across all temperatures (pop: F_1_ = 14.597, *P* < 0.001; temp: F_3_ = 1.118, *P* = 0.351; Table 1). Hematocrit significantly differed across temperature treatments and between populations (pop: F_1_ = 15.564, P < 0.001; temp: F_3_ = 4.530, P = 0.006; Table 1). Potassium levels significantly increased across temperatures, from 2.57 to 3.72 mmol L^−1^ and 2.46 to 4.30 mmol L^−1^ in Chilliwack and Shuswap Chinook salmon, respectively (temp: F_3_ = 4.086, P = 0.010; Table 1). Sodium levels were significantly higher in Chilliwack Chinook salmon than Shuswap Chinook salmon, but there was no effect of temperature treatment (pop: F_1_ = 6.713_,_ P = 0.012; temp: F_3_ = 0.113, P = 0.952). Plasma chloride, osmolality and glucose sampled at the 1 h recovery post MMR_18h_ were not significantly different between populations or across temperatures (*P* = 0.238–0.649; Table 1).

### Population differences and effects of warming on cellular processes

Lactate concentrations sampled 1 h after the exhaustive chase generally increased with test temperature in both the cardiac and white muscle. In the heart, there was a significant effect of test temperature on lactate concentrations (F_3_ = 7.40, P < 0.001; Fig. 7A, Supplementary Table S4). For both populations, the 21 and 24°C exposed fish had higher cardiac lactate concentrations than individuals exposed to 12°C (Fig. 7A, Supplementary Table S4). There was a significant interactive effect of population and test temperature on lactate concentrations in the white muscle (F_2_ = 4.36, *P* = 0.0175), where lactate concentrations were significantly higher in Shuswap Chinook salmon compared with Chilliwack salmon tested at 18°C (P = 0.0187; Fig. 7B, Supplementary Table S4). White muscle lactate concentrations were also significantly higher at 24°C *vs*. 12°C tested Shuswap Chinook salmon (*P* = 0.0238; Fig. 7B, Supplementary Table S4).

**Figure 8:**
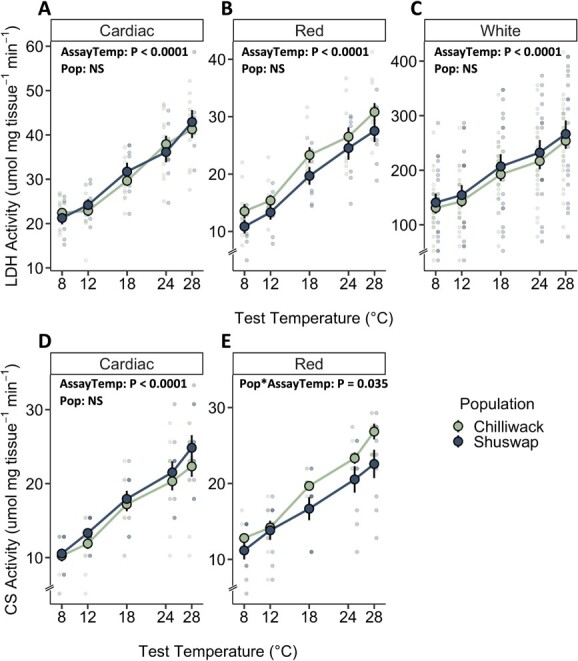
LDH and CS activity in cardiac and skeletal muscle after 1 h recovery in Chilliwack (green symbols) and Shuswap (blue symbols) Chinook salmon. LDH activity (μmol mg tissue^−1^ min^−1^) across assay temperatures (8, 12, 18, 24, 28°C) is measured from **i**ndividuals acclimated to 12°C from (A) heart, (B) red muscle and (C) white muscle and CS activity (μmol mg tissue^−1^ min^−1^) across assay temperature (8, 12, 18, 25, 28°C) is measured from individuals acclimated to 12°C from (D) heart and (E) red muscle. Values are presented as mean activity levels ± SEM and faded individual data points represent values from individual fish, with associated population color. Significant effect terms (assay temperature (AssayTemp), population (Pop)) (ANOVA; Supplementary Table S4) denote statistical results for each tissue. Note the difference in y-axis scale.

In the cardiac, white and red muscle, LDH activity increased with assay temperature in both populations (cardiac: χ^2^_4_ = 494.58, P < 0.0001; white: χ^2^_4_ = 729.30, P < 0.0001; red: χ^2^_4_ = 1310.65; P < 0.0001; Fig. 8A-C, Supplementary Table S4). However, population origin had no effect on LDH activity in cardiac, white, or red tissues (Fig. 8A-C, Supplementary Table S4). In red muscle, CS activity increased with assay temperature in both populations (χ^2^_4_ = 558.71, *P* < 0.001), and enzyme activity appears to increase more quickly with assay temperature in Chilliwack than Shuswap Chinook salmon (Pop^*^AssayTemp, χ^2^_4_ = 10.35, P = 0.035; Fig. 8E, Supplementary Table S4). In cardiac tissue, CS activity increased with increasing assay temperature (χ^2^_4_ = 976.25, P < 0.0001), but in contrast, there was no effect or interactive effect of population (Pop: χ^2^_1_ = 2.85, P = 0.10; Pop^*^Temp χ^2^_2_ = 0.05, P = 0.95; Fig. 8D, Supplementary Table S4).

## Discussion

In this study, we compared intraspecific differences in physiological performances and thermal performance in two populations of Chinook salmon from the Fraser River in British Columbia, Canada. Shuswap Chinook salmon are an interior summer-run population that enters the Fraser River earlier and historically encounters a warmer, longer, more challenging migration whereas Chilliwack Chinook salmon are a coastal fall-run population and enter the river later and encounter a shorter, and historically cooler, migration. We found that Shuswap Chinook salmon were generally more tolerant to high temperatures than Chilliwack Chinook salmon as indicated by better survival rates, wider AAS breadth, and quicker recovery at higher temperatures. However, both populations currently encounter temperatures that approach their upper thermal limits at this pre-spawning life stage, which suggests they may not have adapted at a pace that has kept up with warming.

### Coastal and interior Chinook salmon differ in thermal performance

Our findings indicate that migration history (temperature and physical challenges) plays an important role in population thermal performance and physiological capacities. Intraspecific variability is not uncommon in other salmonids (Lee *et al.,* 2003b; Eliason *et al.,* 2011; Chen *et al.,* 2013, 2015, 2018; Stitt *et al.,* 2014; Verhille *et al.,* 2016; Whitney *et al.,* 2016; Poletto *et al.,* 2017; Abe *et al.,* 2019; Anttila *et al.,* 2019; Zillig *et al.,* 2021; Anlauf-Dunn *et al.,* 2022; Zillig *et al.,* 2022) and in other fish species such as killifish and Atlantic cod (Fangue *et al.,* 2006; Lucassen *et al.,* 2006). Our findings complement previous work in adult sockeye salmon and egg/embryo Chinook salmon from coastal *vs*. interior populations (Beacham and Murray, 1989; Beacham and Withler, 1991; Eliason *et al.,* 2011). Populations with longer and more physically challenging spawning migrations have adapted to their up-river migration conditions with larger somatic energy reserves at the onset of migration, smaller gonadal investment, higher AAS and Topt_AAS_ and greater heart performance as indicated by larger RVM, improved coronary supply, elevated heart rate and greater SERCA activity compared with short-migrating conspecifics (Crossin *et al.,* 2004; Eliason *et al.,* 2011; Cooke *et al.,* 2012; Anttila *et al.,* 2019). Indeed, in this study male Shuswap Chinook salmon had smaller gonadal investment than Chilliwack Chinook salmon (5.3 vs 7.1 GSI%), and while the opposite appeared to be the case for females, sample sizes were likely too small to detect differences and populations may have not been equally at the same level of sexual maturity, with loose eggs observed in the Shuswap but not Chilliwack female fish (pers. obs). However, we did not see a difference in RVM between populations. Nevertheless, our study does not distinguish whether differences in performance metrics are necessarily due to adaptation or acclimation to thermal regimes and/or physical challenges, particularly since the fish were collected near their spawning grounds.

**Table 1:** Population-specific blood chemistry parameters in Chinook salmon (*O. tshawytscha*) 1 h post-MMR_18h_ (chase and air exposure) acclimated to 12°C and tested at 12, 18, 21 and 24°C.

Physiological variable	Population	12 °C	18°C	21°C	24°C	Pop		Temp		Pop ^*^ temp	
						F_(df)_	*P*	F_(df)_	*P*	F_(df)_	*P*
Lactate (mmol L^−1^)	ChilliwackShuswap	14.40 ± 2.92^a^ (14)12.29 ± 2.31^a^ (9)	17.25 ± 3.25^b^ (13)17.66 ± 3.41^b^ (10)	20.52 ± 2.06^b^ (8)17.77 ± 3.54^b^ (9)	NA15.60 ± 1.57^ab^ (3)	3.189_1_	0.079	12.761_3_	**<0.001**	1.640_2_	0.203
Glucose (mmol L^−1^)	ChilliwackShuswap	6.11 ± 1.81 (14)7.01 ± 2.43 (9)	6.74 ± 2.42 (13)7.16 ± 4.04 (10)	6.41 ± 1.35 (8)6.00 ± 1.73 (9)	NA5.01 ± 0.17 (3)	0.346_1_	0.559	0.818_3_	0.489	0.352_2_	0.705
Testosterone (ng ml^−1^)—male	ChilliwackShuswap	10.13 ± 5.70^*^ (13)18.48 ± 13.72^a^^*^ (5)	10.10 ± 8.47 (11)5.41 ± 1.12^b^ (7)	5.18 ± 1.51 (8)4.42 ± 0.51^b^ (7)	NA3.28 ± 0.93^b^ (3)	0.113_1_	0.739	4.595_3_	**0.007**	4.278_2_	**0.020**
Estradiol (ng ml^−1^)—male	ChilliwackShuswap	0.65 ± 0.11 (13)0.50 ± 0.04 (5)	0.63 ± 0.10 (11)0.45 ± 0.02 (7)	0.58 ± 0.09 (8)0.47 ± 0.10 (7)	NA0.42 ± 0.23 (3)	26.022_1_	**<0.001**	1.000_3_	0.401	0.441_2_	0.646
Cortisol (ng ml^−1^)—male	ChilliwackShuswap	576.87 ± 291.01 (13)192.26 ± 74.11 (5)	593.25 ± 214.31 (11)407.75 ± 192.13 (7)	460.22 ± 109.24 (8)327.30 ± 138.62 (7)	NA194.31 ± 110.73 (3)	14.597_1_	**<0.001**	1.118_3_	0.351	1.573_2_	0.218
Hematocrit (%)	ChilliwackShuswap	58.00 ± 6.73^a^ (14)52.00 ± 8.43^a^ (9)	62.23 ± 6.76^ab^ (13)56.20 ± 6.29^ab^ (10)	68.75 ± 6.56^b^ (8)57.00 ± 10.51^b^ (9)	NA62.67 ± 8.02^ab^ (3)	15.564_1_	**<0.001**	4.530_3_	**0.006**	0.882_2_	0.419
Sodium, Na^+^ (mmol L^−1^)	ChilliwackShuswap	151.76 ± 17.98 (14)139.58 ± 19.21 (9)	150.63 ± 9.73 (13)146.83 ± 9.70 (10)	153.39 ± 14.42 (8)140.93 ± 10.50 (9)	NA142.37 ± 3.21 (3)	6.713_1_	**0.012**	0.113_3_	0.952	0.663_2_	0.519
Chloride, Cl^−^ (mmol L^−1^)	ChilliwackShuswap	121.36 ± 14.21 (14)116.20 ± 17.38 (9)	116.14 ± 5.43 (13)121.75 ± 9.30 (10)	112.75 ± 8.96 (8)117.39 ± 13.13 (9)	NA122.17 ± 5.97 (3)	0.248_1_	0.621	0.551_3_	0.649	1.370_2_	0.262
Potassium, K^+^ (mmol L^−1^)	ChilliwackShuswap	2.57 ± 0.95^a^ (14)2.46 ± 1.36^a^ (9)	3.10 ± 1.31^ab^ (13)3.09 ± 0.88^ab^ (10)	3.72 ± 1.64^b^ (8)3.91 ± 1.75^b^ (9)	NA4.30 ± 0.75^ab^ (3)	0.0004_1_	0.985	4.086_3_	**0.010**	0.066_2_	0.936
Osmolality (mOsm kg^−1^)	ChilliwackShuswap	311.21 ± 26.46 (14)300.78 ± 28.40 (9)	313.54 ± 9.30 (13)314.10 ± 9.45 (10)	313.75 ± 10.86 (8)306.22 ± 15.96 (9)	NA312.00 ± 4.58 (3)	1.418_1_	0.238	0.597_3_	0.620	0.521_2_	0.597

Inside parentheses are sample size.

Represented are means and standard deviation values for each treatment group, and ANOVA results. Physiological variables that were significantly affected by population, temperature or the interaction of population and temperature are bolded, letters indicate significant post hoc HD results across temperatures and asterisks indicate significant post hoc HD results across populations (P < 0.05). Temp, temperature; Pop, population; df, degrees of freedom.

We found similar aerobic capacities but different thermal breadths between these Chinook salmon populations. The AAS values at Topt_AAS_ (9.6 and 10.0 mg O_2_ kg^−1^ min^−1^ in Chilliwack and Shuswap, respectively) correspond with the AAS of other adult salmon (chum 10.3–10.7 mg O_2_ min^−1^ kg^−1^ (Abe *et al.,* 2019); pink 7.7–18.3 mg O_2_ min^−1^ kg^−1^ (Clark *et al.,* 2011); rainbow trout 9.2 mg O_2_ min^−1^ kg^−1^ (Chen *et al.,* 2015); sockeye 7.7–11.8 mg O_2_ min^−1^ kg^−1^ (Eliason *et al.,* 2011)) and these AAS values are also comparable when only accounting for survivors (Fig. 4E). Notably, MMR and AAS were consistent whether assessed 1 h or 18 h after acute thermal exposure, demonstrating rapid thermal compensation (Supplementary Table S3). Interestingly, the MMR values measured post-chase underestimated AAS in pre-spawning Chinook salmon since 39% of fish demonstrated their highest MO_2_ value during overnight recovery. This demonstrates the importance of examining all MO_2_ slopes and using careful interpretation when estimating MMR (Killen *et al.,* 2017). To our knowledge, this study is the first to measure AAS in maturing salmon obtained near their spawning grounds. The optimal upper thermal windows (defined by Topt_AAS_ and upper T_pejus_) of 14.75 to 18.70 ± 2.96°C (SD) and 15.30 to 20.15 ± 3.70°C in the Chilliwack and Shuswap populations demonstrate a wider thermal breadth in Shuswap salmon. Generally, these temperature ranges are consistent with previous findings for migrating adult Chinook salmon thermal limits, which wait at river mouths and slow their migration when river temperatures are too warm (20–23.9°C Columbia River; 19–21°C Sacramento River) (Fish and Hanavan, 1948; Hallock *et al.,* 1970; Richter and Kolmes, 2005; Goniea *et al.,* 2006; Keefer *et al.,* 2018; for review see McCullough, 2001). We did not find major differences in Topt_AAS_ between the populations and this might be because we did not test fish within the thermal range that is likely to be “optimal” (14–17°C) as measured in sockeye and coho (*O. kisutch*) salmon (Eliason *et al.,* 2011; Kraskura *et al.,* 2021) and instead focused on testing fish at the supraoptimal temperatures to define temperatures where performance would collapse.

We took an integrative approach across biological levels of organization to identify mechanisms for differences in physiological and thermal performance by sampling tissue from fish after 1 h of recovery following MMR_18h_. While we expected to find greater capacity for aerobic and anaerobic metabolism in Shuswap Chinook salmon (e.g. higher activity levels of CS and LDH), we saw no population differences in CS or LDH activity across assay temperatures that are in line with the AAS findings. However, high inter-individual variability in enzyme activities indicated individual differences in physiological performance. Indeed, individuals may differ in their performance and thermal capacity via cellular plasticity and local adaptation (Taylor, 1991). Previous work has found population-specific and sex-specific differences in the activity or density of enzymes associated with the metabolic or cardiac function of salmon (Rodnick *et al.,* 2008; Eliason *et al.,* 2011; Anttila *et al.,* 2019; Little *et al.,* 2020b), which points to the utility of cellular level investigation and variety of mechanisms that may underlie differences in organismal performance.

### Recovery is impaired in both populations at projected river temperatures

Recovery is critical for salmon during their once-in-a-lifetime spawning migration and inability to recover may lead to premature death and failure to complete migration (Makiguchi *et al.,* 2011; Burnett *et al.,* 2014a). It is suggested that salmon need 80–90% of AAS to complete their spawning migration (Farrell *et al.,* 2008; Eliason *et al.,* 2011) and salmon need to recover to 50% of their AAS to repeat their swim performance (Farrell *et al.,* 1998, 2003; Jain *et al.,* 1998; Jain and Farrell, 2003; MacNutt *et al.,* 2006). In accordance with our hypothesis, Shuswap salmon had improved recovery capacities at higher temperatures compared with Chilliwack salmon. Chilliwack salmon tested at 18°C required an extra 1 h to reach 50% MMR compared with Shuswap salmon (Supplementary Table S3). Further, Chinook salmon tested at 21°C were only at 15% (Chilliwack) and 43% (Shuswap) AAS after 1 h of recovery. Given that salmon on the spawning grounds are often in their last hours to days of life, this time lost to recovery is significant.

Temperatures above 21°C hindered recovery for both populations, as exhibited by lactate and potassium levels. Previous studies identified “threshold values” for plasma lactate, where levels must decrease below a threshold of 10–13 mmol L^−1^ before an individual can repeat swim performance in the lab (sockeye salmon: Farrell *et al.,* 1998; rainbow trout: Jain and Farrell, 2003). In the wild, ocean telemetry tracking studies have found that sockeye salmon will perish if plasma lactate exceeds 18–20 mmol L^−1^ during capture (Crossin *et al.,* 2009). After 1 h of recovery following exhaustive exercise, Chilliwack Chinook salmon had higher plasma lactate levels than Shuswap Chinook salmon at each temperature, indicating Chilliwack salmon utilized anaerobic metabolism to a greater extent in response to the chase protocol and/or had impaired ability to clear lactate. However, these populations had similar tissue LDH activities, indicating there was no difference in lactate clearance. Plasma lactate exceeded the above thresholds and suggests that Chinook salmon are either more tolerant to high lactate levels and/or are past their threshold and would be unable to repeat swim at the time of sampling (Farrell *et al.,* 1998; Jain and Farrell, 2003). Additionally, hyperkalemia (5 mmol L^−1^ K^+^ perfusate) reduces cardiac output by 30% (Hanson *et al*. (2006). As indicated by cardiac lactate and plasma potassium levels here, temperatures beyond 21°C could have compromised cardiac function in both populations.

### Management implications

The Fraser River is the greatest producer of Pacific salmon in Canada and supports a major share of Canadian Chinook salmon populations (Northcote and Atagi, 1997; Beacham *et al.,* 2002). However, the Fraser River and its tributaries are warming at an alarming rate and current maximum temperatures (daily means) during the upriver migration to the spawning grounds are 21.5°C for Chilliwack and 22.8°C for Shuswap Chinook salmon (Fraser River EWatch, 2021). These temperatures exceed the upper T_pejus_ of these two populations of pre-spawning adult Chinook salmon (Chilliwack, 18.7°C; and Shuswap, 20.2°C; Fig. 5), which mark the maximum temperature below which fish are predicted to perform near optimally. Accordingly, Chilliwack and Shuswap Chinook salmon may have a functional warming tolerance (max environmental temperature-upper T_pejus_) (Anlauf-Dunn *et al.,* 2022) of 2.8 and 2.6°C, respectively, *en route* to the spawning grounds. It is evident that current and in the near future, Fraser River temperatures are approaching the functional limits of Chinook salmon.

Indeed, here we included both the aerobic scopes of all fish (Fig. 4C, D) and survivors only (Fig. 4E, F) to prevent misinterpretation of the results (Patterson *et al.,* 2016). While it is uncommon to calculate aerobic scope using mortalities because the recorded data from moribund fish does not represent SMR or MMR, a mortality indicates that the individual has zero aerobic capacity and would not sufficiently swim upstream or successfully spawn (Eliason and Farrell, 2016). Mortalities occurred across high temperatures (18, 21, 24°C) in our experiment and discounting these mortalities would greatly overestimate the aerobic capacity of a population. As the purpose of this study is in part, to provide the science to managers and stakeholders about the ability of these Chinook salmon to perform during their final life stage, it is essential that we include all individuals in the experiment to accurately estimate the aerobic capacity of each population.

Acclimation to warming by chronic thermal exposure can increase AAS by depressing SMR (Seebacher *et al.,* 2015; Sandblom *et al.,* 2016) and therefore might increase the energy available to successfully migrate and spawn. We measured the physiological performance of salmon in response to short-term, acute temperature exposures. In the wild, pre-spawning salmon experience big temperature changes in this acute timeframe because they are generally restricted to their spawning grounds with limited space, thermal heterogeneity and time because they are senescing (e.g. Donaldson *et al.,* 2009). This contrasts with the 2–4-week chronic acclimation studies that are more typical in a laboratory. The inter-individual variability that we observed in physiological performance across biological levels within populations suggests differences in acclimation capacity and requires further investigation. For example, female salmon are more vulnerable to secondary stressors than male salmon and are returning to the spawning grounds in lower numbers than historically (Hinch *et al.,* 2021). While our study did not have adequate numbers to comprehensively compare sex-specific performance, the premature mortalities in the few female individuals we measured leave room for further inquiry into sex-specific vulnerabilities.

## Conclusions

Our work demonstrates that pre-spawning adult Chinook salmon are vulnerable to warming river temperatures. These populations, being separated by several months in migration onset and hundred kilometers of migratory distance, displayed intraspecific and inter-individual variability in physiological performance at acute but temporally relevant thermal exposures scaling from the cell to population level. Thermal performance paralleled migratory history: Coastal Chilliwack Chinook salmon that migrate during cooler seasons are less tolerant to high temperatures than Interior Shuswap Chinook salmon that migrate during warmer seasons. Even still, based on physiological thermal performance and current temperature exposure risks, these populations have roughly equivalent vulnerability to river warming. Both populations currently experience river temperatures that are almost 3°C warmer than their functional warming tolerance. Maintaining a diverse portfolio of physiological traits within and between populations can increase resilience and support the variation needed for adaptive change (Zillig *et al.,* 2021). Therefore, protecting physiological diversity is essential to Pacific salmon conservation (Cordoleani *et al.,* 2021).

## Funding

This work was supported by the National Science Foundation [Graduate Research Fellowship Support to JCV]; Natural Sciences and Engineering Research Council of Canada [Collaborative Research Development Grant and Discovery Grant to SGH]; the University of California, Santa Barbara [Faculty Research Grant to EJE]; Department of Fisheries and Oceans Canada [Environmental Watch Program to DAP]; and the Swedish Research Council [Vetenskapsrådet, 2018-00516 to AE].

## Author Contributions

Conceptualization, supervision, project administration and funding acquisition: EJE, SGH, DAP, SJC. Methodology: JCV, BH, AE; Formal analysis, data curation: JCV; Writing: JCV, EJE; Editing: All authors contributed to editing.

## Conflicts of Interest

The authors have no conflicts to declare.

## Data Availability Statement

Data is available on Dryad (Van Wert *et al.,* 2023): https://doi.org/10.25349/D9490W.

## Supplementary Material

Web_Material_coad022
