# Supplementary material for: Population variability in thermal performance of pre-spawning adult Chinook salmon
Source: Conserv Physiol. 2023 May 3;11(1):coad022. doi: 10.1093/conphys/coad022 (PMC10157787; doi:10.1093/conphys/coad022)
Supplement: Web_Material_coad022 [file web_material_coad022.zip › vanwert-supplementary-materials.pdf]

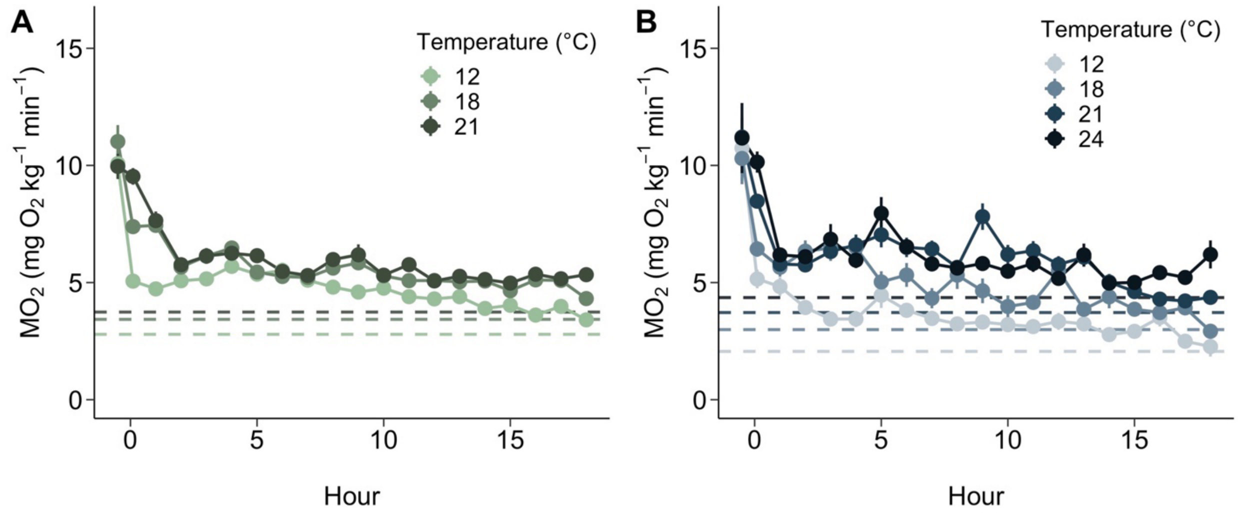

Figure S1. Oxygen consumption during recovery. Raw oxygen consumption values during the recovery period post MMR_{1h} for (A) Chilliwack (green symbols) and (B) Shuswap (blue symbols) Chinook salmon (*O. tshawytscha*) acclimated to 12°C and tested at 12, 18, and 21°C in all Chinook salmon as well as 24°C in Shuswap Chinook salmon. Horizontal dashed lines represent mean resting metabolic rate (RMR) values for each treatment. Color gradient corresponds to temperature treatment, increasing in darkness with temperature. Values are expressed as mean ± SEM. MMR_{1h} and RMR are corrected using the metabolic scaling coefficient of 0.58 and 0.67, respectively.

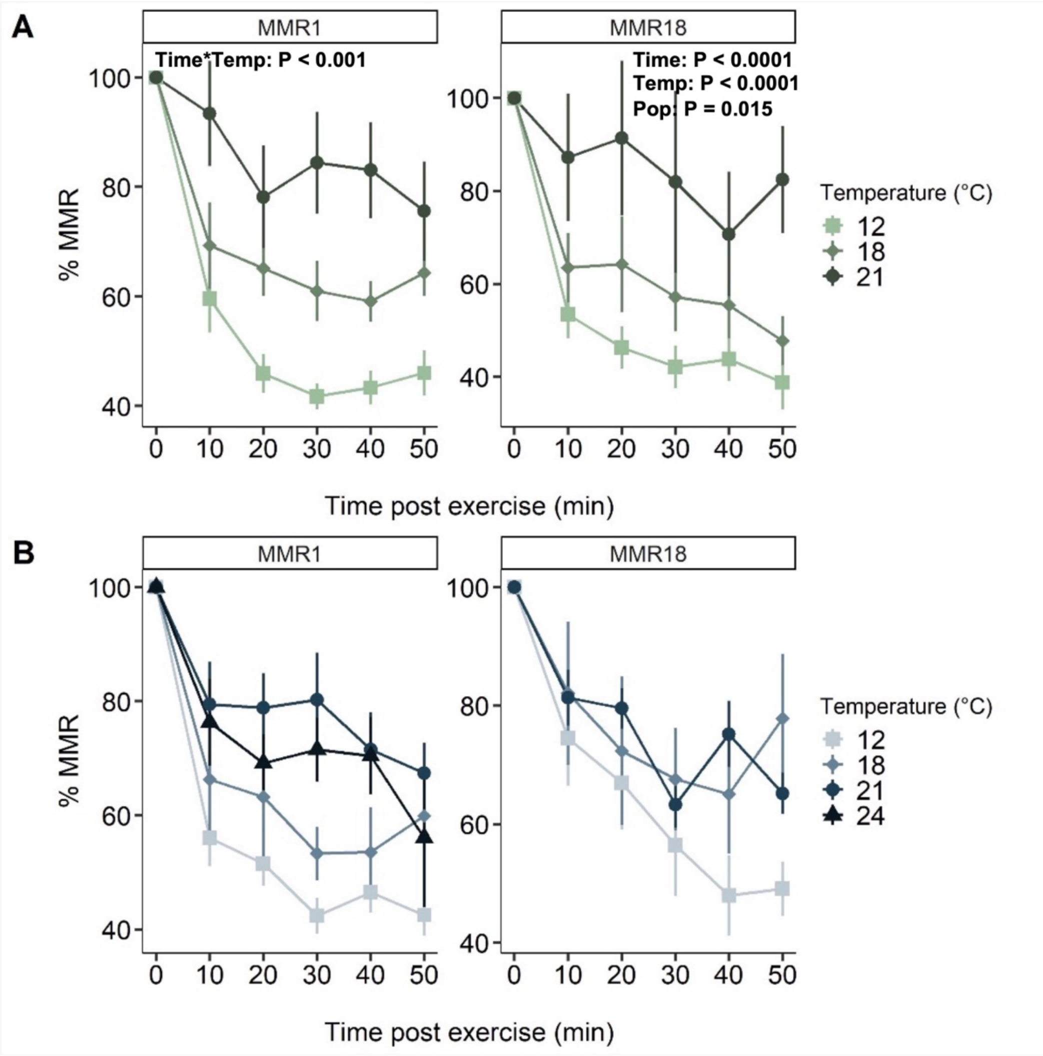

Figure S2. Short term recovery following exhaustive exercise in Chinook salmon (*O. tshawytscha*). Short-term recovery measured as percent of MMR during the 50 min of recovery following MMR_{1h} (first column) and MMR_{18h} (second column) for (A) Chilliwack (green symbols) and (B) Shuswap (blue symbols) Chinook salmon acclimated to 12°C and tested at 12, 18, 21 and 24°C. Values are pooled every 10 min as mean percent of MMR ± SEM. Significant two-way interaction (timepoint (Time) * test temperature (Temp)) (ANOVA) or significant effect of each effect term (timepoint (Time), test temperature (Temp), population (Pop)) (ANOVA) denote statistical results for each column (MMR_{1h} or MMR_{18h}). Note that there are no recovery values for 24°C Chilliwack Chinook salmon at the 18 h exhaustive exercise because of full mortality following the first exhaustive exercise recovery period and no 24°C Shuswap Chinook salmon at the 18 h exhaustive exercise because of low sample size (N = 2).

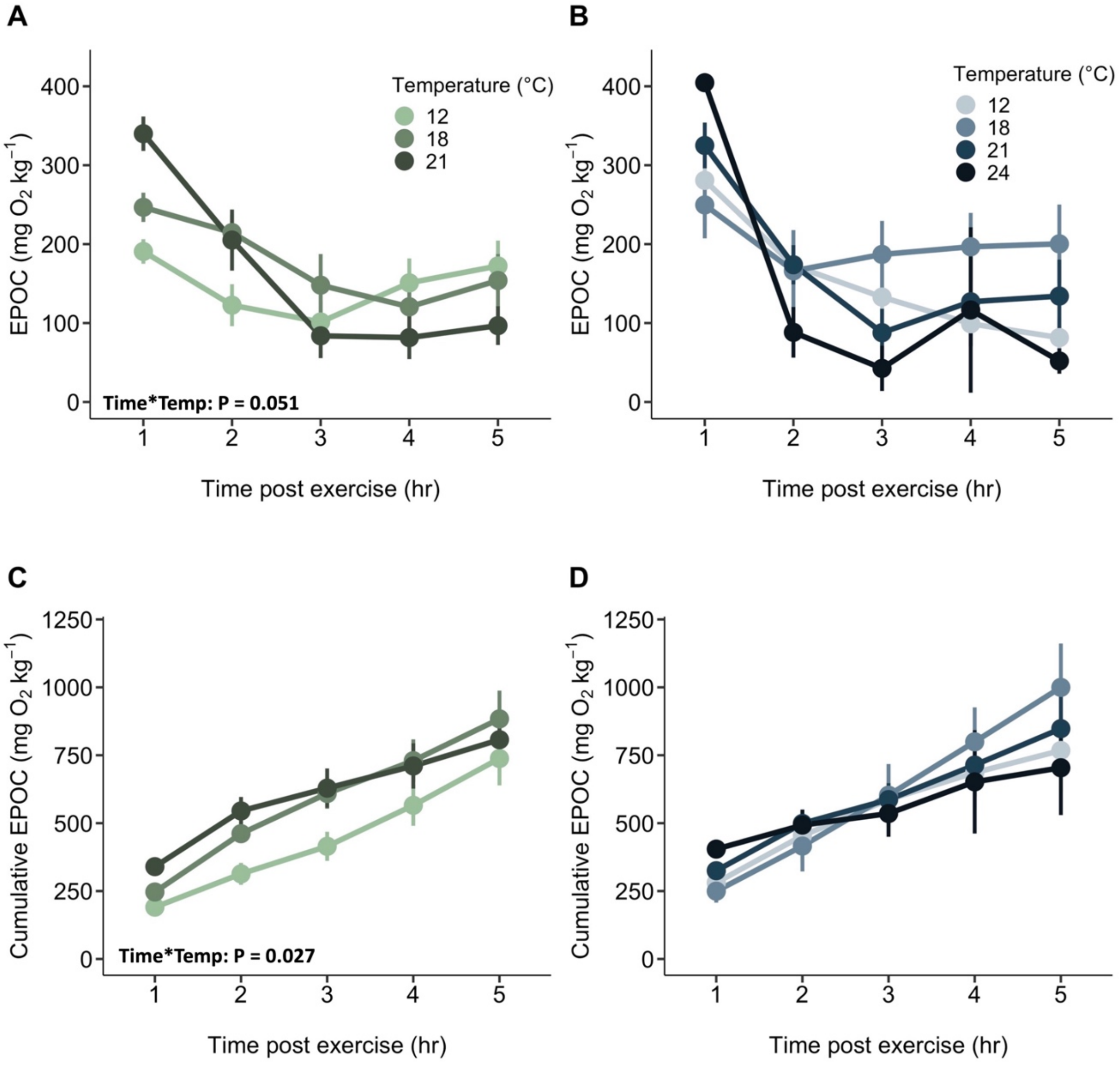

Figure S3. Excess post exercise oxygen consumption (EPOC) in Chinook salmon (*O. tshawytscha*). Hourly EPOC (A, B) and cumulative EPOC (C, D) for the first five hours following MMR_{1h} in Chilliwack (green symbols; A, C) and Shuswap (blue symbols; B, D) Chinook salmon held at 12°C and acutely exposed to different temperatures (12, 18, 21, 24°C). Values are expressed as mean ± SEM. Significant results for populations are denoted in column 1, with a two-way interaction (timepoint (Time) * test temperature (Temp)) (ANOVA) for hourly EPOC (A, B) and cumulative EPOC (C, D).

Table S1. Summary of Chinook salmon (*O. tshawytscha*) morphometrics from Chilliwack and Shuswap populations. Fish at the 12°C assigned temperature were transferred 1 h before the exercise test, 18°C 4 h, 21°C 5.5 h, and 24°C 8 h before the exercise test. The values are expressed as mean ± SEM.

	Chilliwack					Shuswap				
	**12°C**	**18°C**	**21°C**	**24°C**	**Overall**	**12°C**	**18°C**	**21°C**	**24°C**	**Overall**
**Fork Length (mm)**	633.6 ± 24.2	625.6 ± 28.2	636.8 ± 26.8	626.5 ± 56.4	631.6 ± 14.3	687.2 ± 26.1	673.9 ± 20.9	681.6 ± 33.8	645.9 ± 15.9	673.2 ± 12.6
**Body mass (kg)**	3.32 ± 0.44	3.19 ± 0.47	3.39 ± 0.45	3.42 ± 1.03	3.31 ± 0.25	3.84 ± 0.41	3.55 ± 0.30	3.72 ± 0.55	3.07 ± 0.37	3.56 ± 0.21
**Male (N)**	13	12	13	4	42	5	7	7	7	26
**Female (N)**	1	2	2	0	5	4	4	3	1	12
**Total N**	14	14	15	4	47	9	11	10	8	38

Table S2. Mortality rates for Chilliwack and Shuswap Chinook salmon (*O. tshawytscha*). Mortality includes any mortality that occurred during the experiment (from the start of the acute temperature exposure to 1 h after the second MMR measurement). NA indicates no fish were run for that treatment.

Population	Temperature (°C)	Total Experimented		Total Mortality		Mortality (%)		Total mortality (%)
		Male	Female	Male	Female	Male	Female	
Chilliwack	12	13	1	0	0	0.0	0.0	0.0
	18	12	2	1	0	8.3	0.0	7.1
	21	13	2	5	2	38.5	100.0	46.7
	24	4	0	4	NA	100.0	NA	100.0
Shuswap	12	5	4	0	0	0.0	0.0	0.0
	18	7	4	0	1	0.0	25.0	9.1
	21	7	3	0	1	0.0	33.3	10.0
	24	7	1	4	1	57.1	100.0	62.5

Table S3. Summary of metabolic performances and recovery rates in Chilliwack and Shuswap Chinook salmon (*O. tshawytscha*). Values are expressed as mean ± SEM and are corrected to a common body mass of 3.5 kg using the metabolic scaling coefficient of 0.58 for MMR, 0.67 for RMR, AAS as MMR-RMR (mg O_2 kg $^{-1}$min^{-1}), FAS as MMR/RMR (unit-less), and in min to 50% MMR_{1h} (recMMR50). Temperature treatments with different letters within a population are statistically different from each other, and an asterisk indicates a statistically significant difference from the other population within the temperature treatment ($P < 0.05$); significant p-values are bolded for emphasis. NA indicates no salmon were completed for the metric.

				**Mean ± SEM**		**Pop**		**Temp**		**Pop*Temp**	
**Performance Metric**	Temperature (°C)	N Chilliwack	N Shuswap	Chilliwack	Shuswap	F_{df}	P-value	F_{df}	P-value	F_{df}	P-value
Resting Metabolic Rate (mg O_2 kg^{-1}min^{-1})	12	14	9	2.70 ± 0.24^{a}	2.06 ± 0.21^{a}	3.940_1	0.053	12.886_3	**< 0.001**	0.784_2	0.462
	18	11	8	3.43 ± 0.23^{b}	2.99 ± 0.39^{b}						
	21	8	9	3.74 ± 0.20^{b}	3.72 ± 0.10^{b}						
	24	NA	2	NA	4.36 ± 0.12^{b}						
Maximum Metabolic Rate 1h (mg O_2 kg^{-1}min^{-1})	12	14	9	10.07 ± 0.61	10.74 ± 1.37	1.124_1	0.293	0.245_3	0.865	1.141_3	0.339
	18	14	11	11.02 ± 0.70	10.30 ± 1.10						
	21	12	10	9.96 ± 0.54	11.21 ± 0.79						
	24	4	6	7.99 ± 1.33	11.17 ± 1.50						
Maximum Metabolic Rate 18 h (mg O_2 kg^{-1}min^{-1})	12	13	8	10.64 ± 0.66	8.62 ± 1.32	4.055_1	**0.049**	0.410_3	0.746	0.288_2	0.751
	18	13	7	11.06 ± 0.88	9.16 ± 1.37						
	21	5	8	9.18 ± 1.00	8.65 ± 0.63						
	24	NA	3	NA	8.31 ± 1.02						
Maximum Metabolic Rate Overall (mg O_2 kg^{-1}min^{-1})	12	14	9	11.26 ± 0.55	11.41 ± 1.33	0.371_1	0.545	1.938_3	0.131	1.872_3	0.142
	18	14	11	12.80 ± 0.60	11.44 ± 1.08						
	21	12	10	11.44 ± 0.59	12.81 ± 0.68						
	24	4	6	7.99 ± 1.33	11.17 ± 1.50						
Absolute Aerobic Scope (mg O_2 kg^{-1}min^{-1}) [with 0's for mortalities if died to this timepoint]	12	14	9	8.56 ± 0.51^{a}	9.35 ± 1.14^{a}	6.436_1	**0.013**	16.096_3	**< 0.001**	3.396_3	**0.023**
	18	11	9	8.96 ± 0.58^{a}	8.02 ± 1.19^{a}						
	21	15	9	$4.10 \pm 1.11^{b*}$	$9.14 \pm 0.79^{a*}$						
	24	4	7	0^{b}	2.73 ± 1.76^{b}						
Factorial Aerobic Scope	12	14	9	4.45 ± 0.36^{a}	5.54 ± 0.33^{a}	10.822_1	**0.002**	32.343_3	**< 0.001**	1.516_3	0.218
	18	11	9	3.76 ± 0.30^{b}	3.86 ± 0.59^{b}						

[with 0's for mortalities if died to this timepoint]	21	15	9	1.65 ± 0.43^{c}	3.49 ± 0.24^{c}						
	24	4	7	0^{d}	0.91 ± 0.59^{d}						
Absolute Aerobic Scope (mg O_2 $kg^{-1}min^{-1}$) [survivors only]	12	14	9	8.56 ± 0.51	9.35 ± 1.14						
	18	11	8	8.96 ± 0.58	9.02 ± 0.72	1.500_1	0.226	0.356_2	0.702	0.393_2	0.677
	21	8	9	7.69 ± 0.84	9.14 ± 0.79						
	24	NA	NA	NA	NA						
Factorial Aerobic Scope [survivors only]	12	14	9	4.45 ± 0.36^{a}	5.54 ± 0.33^{a}						
	18	11	8	3.76 ± 0.30^{b}	4.34 ± 0.38^{b}	6.968_1	**0.011**	12.871_2	**< 0.001**	0.596_2	0.555
	21	8	9	3.09 ± 0.25^{b}	3.49 ± 0.24^{b}						
	24	NA	NA	NA	NA						
Time to 50 % MMR ($recMMR_{50}$) (min)	12	14	9	23.86 ± 2.03^{a}	32.22 ± 4.13^{a}						
	18	13	9	$107.00 \pm 22.16^{b*}$	$47.00 \pm 15.90^{ab*}$	1.587_1	0.212	10.531_3	**< 0.001**	3.060_2	**0.054**
	21	10	10	107.20 ± 18.45^{b}	87.30 ± 16.18^{b}						
	24	NA	3	NA	143.00 ± 75.11^{b}						

Table S4. Statistical (mixed-model ANOVA) parameters for activities of lactate dehydrogenase (LDH) and citrate synthase (CS) and concentrations of lactate in cardiac, red, and white muscle from Chinook salmon (*O. tshawytscha*). N represents range of samples from population (LDH, CS) or temperature treatments within a population (lactate).

Tissue	Analysis	Parameter	F or χ^2	d.f.	P-value
Red muscle	LDH (N = 8 – 11)	Pop	2.09	1	0.15
		AssayTemp	1310.65	4	**< 0.0001**
		Pop*AssayTemp	3.43	4	0.49
	CS (N = 9 – 12)	Pop.	3.61	1	0.057
		AssayTemp	558.71	4	**< 0.0001**
		Pop*AssayTemp	10.35	4	**0.035**
White muscle	LDH (N = 16 – 24)	Pop	0.83	1	0.36
		AssayTemp	729.30	4	**< 0.0001**
		Pop*AssayTemp	1.76	4	0.78
	Lactate (N = 8 – 12; 3 24°C)	Pop	0.57	1	0.45
		Temp	1.81	3	0.16
		Pop*Temp	4.36	2	**0.018**
Cardiac muscle	LDH (N = 8 – 13)	Pop	0.04	1	0.85
		AssayTemp	494.58	4	**< 0.0001**
		Pop*AssayTemp	5.98	4	0.20
	CS (N = 10 – 14)	Pop	0.95	1	0.33
		AssayTemp	976.25	4	**< 0.0001**
		Pop*AssayTemp	3.04	4	0.55
	Lactate (N = 7 – 12; 3 24°C)	Pop	2.85	1	0.10
		Temp	7.40	3	**< 0.001**
		Pop*Temp	0.05	2	0.95
